# The Basic Helix-Loop-Helix Transcription Factor Family in the Pea Aphid, *Acyrthosiphon pisum*

**DOI:** 10.1673/031.011.8401

**Published:** 2011-07-07

**Authors:** Chun-Wang Dang, Yong Wang, Ke-Ping Chen, Qing Yao, De-Bao Zhang, Min Guo

**Affiliations:** ^1^ Institute of Life Sciences, Jiangsu University, 301 Xuefu Road, Zhenjiang 212013, P. R. China; ^2^ School of Food and Biological Engineering, Jiangsu University, 301 Xuefu Road, Zhenjiang 212013, P. R. China

**Keywords:** blast search, orthologous family, phylogenetic analysis

## Abstract

The basic helix-loop-helix (bHLH) proteins play essential roles in a wide range of developmental processes in higher organisms. bHLH family members have been identified in over 20 organisms, including fruit fly, zebrafish, and human. This study identified 54 bHLH family members in the pea aphid, *Acyrthosiphon pisum* (Harris) (Hemiptera: Aphididae), genome. Phylogenetic analyses revealed that they belong to 37 bHLH families with 21, 13, 9, 1, 9, and 1 members in group A, B, C, D, E, and F, respectively. Through in-group phylogenetic analyses, all of the identified *A. pisum* bHLH members were assigned into their correspondent bHLH families with confidence, among which 51 were defined according to phylogenetic analyses with orthologs from *Drosophila melanogaster* Meigen (Diptera: Drosophilidae), and 3 of them were defined according to phylogenetic analyses with orthologs from *Bombyx mori* L. (Lepidoptera: Bombycidae) and *Tribolium castaneum* (Herbst) (Coleoptera: Tenebrionidae). Analyses on genomic coding regions revealed that the number and average length of introns in *A. pisum* bHLH motifs are higher than those in other insects. The present study provides useful background information for future studies on structure and function of bHLH proteins in the regulation of *A. pisum* development.

## Introduction

The basic helix-loop-helix (bHLH) proteins form a large superfamily of transcription factors that play important roles in a wide range of developmental processes including neurogenesis, myogenesis, hematopoiesis, sex determination, and gut development. The bHLH domain is approximately 60 amino acids long and comprises a DNA-binding basic region (b) and two helices separated by a variable loop region (HLH) (Massari and Murre 2000). The HLH domain promotes dimerization, allowing the formation of homodimeric or heterodimeric complexes between different family members. The two basic domains which are brought together through dimerization bind specific hexanucleotide sequences.

Since the first characterization of the murine bHLH transcription factors E12 and E47 (Murre et al. 1989), Atchley et al. (1999) developed a predictive motif for the bHLH domains based on amino acid frequencies at all positions of 242 bHLH proteins, among which 19 sites were highly conserved in all the organisms. With the completion of genome sequencing projects for an increased number of organisms, over one thousand bHLH family members have been identified in organisms whose genome sequences were available. These include 8 bHLH genes in *Saccharomyces cerevisiae,* 16 in *Amphimedon queenslandica,* 33 in *Hydra magnipapillata,* 33 in *Caenorhabditis elegans,* 104 in *Gallus gallus,* 46 in *Ciona intestinalis,* 50 in *Strongylocentrotus purpuratus,* 51 in *Apis mellifera,* 52 in *Bombyx mori,* 57 in *Daphia pulex,* 59 in *Drosophila melanogaster,* 63 in *Lottia gigantea,* 64 in *Capitella* sp 1, 68 in *Nematodtella vectensis,* 78 in *Branchiostoma floridae,* 87 in *Tetraodon nigroviridis,* 114 in *Mus musculus,* 118 in *Homo sapiens,* 139 in *Brachydanio rerio,* 147 in *Arabidopsis,* and 167 in *Oryza sativa* (Zheng et al. 2009; Li et al. 2006; Satou et al. 2003; Simionato et al. 2007; Toledo-Ortiz et al. 2003; Wang et al. 2007, 2008, 2009).

Based on phylogenetic analyses to the available bHLH proteins, Ledent and Vervoort (2001) defined 44 orthologous families and 6 higher-order groups for bHLH proteins, among which 36 include bHLH from animals only, two have representatives in both yeasts and animals, two are present only in yeast, and four are present only in plants. They named the 44 families according to their first reported names, common abbreviations, or their best-known members of the family. And the higher-order groups were named A, B, C, D, E, and F based on their different DNA-binding properties of these groups. Group A and B include bHLH proteins that bind hexameric DNA sequences referred to as “E boxes” (CANNTG), in which group A binds to CACCTG or CAGCTG and group B binds to CACGTG or CATGTTG (Murre et al. 1989; Van Doren et al. 1991; Dang et al. 1992). Group C corresponds to the family of bHLH proteins known as bHLH-PAS which is about 260–310 amino acids long (Crews 1998). bHLH-PAS proteins bind the core sequence of ACGTG or GCGTG. Group D corresponds to HLH proteins that lack a basic domain. They form inactive heterodimers with group A proteins. Group E corresponds to the family of bHLH proteins which bind preferentially to sequences typical of N boxes (CACGCG or CACGAG). They also contain one additional Orange domain and one WRPW peptide in their carboxyl terminus. Group F corresponds to the family of bHLH proteins that have the COE domain which has an additional domain involved in both dimerization and DNA binding (Ledent and Vervoort 2001).

Ledent et al. (2002) defined 44 families for bHLH proteins from animals only, among which 20, 12, 7, 1, 3, and 1 families are included in groups A, B, C, D, E, and F, respectively. In 2007, it was found that the MyoR family could be expanded into three families, i.e. MyoRa , MyoRb, and Delilah, and the originally separated families, Hairy and E(spl), needed to be combined into one family, H/E(spl), due to insufficient evidence from the phylogenetic analyses (Simionato et al. 2007). Therefore, at present, animal bHLH proteins are classified into 45 families.

The pea aphid, *Acyrthosiphon pisum* (Harris) (Hemiptera: Aphididae), is the primary aphid species used in laboratory and genetic studies. *A. pisum* has been intensively studied as a model for understanding bacterial endosymbiosis, phenotypic plasticity, clonal vs. sexual reproduction, and the development of resistance to pesticides (Wilson et al. 2010; Srinivasan et al. 2010). bHLH proteins are important transcription factors with regulatory functions in various developmental processes in eukaryotes. Identification of bHLH protein members encoded in the *A. pisum* genome will facilitate studies on gene structure and function involved in regulation of *A. pisum* development. However, there have been no reports on identification and characterization of *bHLH* genes in *A. pisum.* In this study, amino acid sequences of 59 *D. melanogaster* Meigen (Diptera: Drosophilidae) bHLH motifs were used to conduct tblastn searches against *A. pisum* genome sequences (http://www.ncbi.nlm.nih.gov/genomeprj/13646) to obtain candidate bHLH members in *A. pisum.* Subsequent examination and analyses led to successful identification of 54 bHLH members in *A. pisum* and definition of orthologous families for them with sufficient confidence. Moreover, it was found that the number and average length of introns in *A. pisum* bHLH motifs are higher than those in other insects. These results provide useful background information for future studies on structure and function of bHLH proteins in the regulation of *A. pisum* development.

## Materials and Methods

### Tblastn searches

Amino acid sequences of 59 *D. melanogaster* bHLH motifs were obtained from the additional files of previous reports (Ledent and Vervoort 2001; Simionato et al. 2007). Each sequence was used as query sequence to perform tblastn searches against the *A. pisum* genome sequences. The expected value (*E*) was set at 10 in order to obtain all bHLH related sequences. The obtained subject sequences were manually examined to keep only one sequence for those that have the same contig number, reading frame, and coding regions; to add the missing amino acids to corresponding sites by EditSeq program (version 5.01) of the DNAStar package; and to find introns within the bHLH motifs. Intron analysis was done using NetGene2 application online (http://www.cbs.dtu.dk/services/NetGene2/).

### Sequence alignment

All sequences that had been improved by the above methods were aligned using MEGA4 (Tamura et al. 2007) built-in ClustalW program (version 4.0) with default settings. Each sequence was examined for their amino acid residues at the 19 conserved sites by manual checking. Sequences with less than nine variations were regarded as potential ApbHLH (*A. pisum* bHLH) members. The sequences which have less than ten conservations were discarded and the rest sequences were aligned again using ClustalW. The aligned ApbHLH motifs were shaded in GeneDoc Multiple Sequence Alignment Editor and Shading Utility (Version 2.6.02) (Nicholas et al. 1997) and copied to rich text file for further annotation.

### Phylogenetic analyses

Phylogenetic analyses to all the identified ApbHLH members were carried out in two steps. First, all obtained ApbHLH motif sequences were used to build neighbor-joining (NJ) distance tree with the 59 *D. melanogaster* bHLH motif sequences using PAUP 4.0 Beta 10 (Swofford 1998) based on a step matrix constructed from Dayhoff PAM 250 distance matrix by R. K. Kuzoff (http://paup.csit.fsu.edu/). Then, each ApbHLH motif sequence was used to conduct in-group phylogenetic analyses (Wang et al. 2007) with *D. melanogaster* bHLH motif sequences. That is, each amino acid sequence of *A. pisum* bHLH motifs was used to construct NJ, maximum parsimony (MP), and maximum likelihood (ML) phylogenetic trees with *D. melanogaster* bHLH family members of the corresponding group, respectively. The NJ trees were bootstrapped with 1000 replicates to provide information about their statistical reliability. MP analysis was performed using heuristic searches and bootstrapped with 100 replicates. ML trees were constructed using TreePuzzle 5.2 (Schmidt et al. 2002) with quartet-puzzling tree-search procedure and 25,000 puzzling steps. Model of substitution was set to the Jones-Taylor-Thornton (Jones et al. 1992). Other parameters were set to default values.

## Results and Discussion

### Identification of ApbHLH members

**Figure 1.**
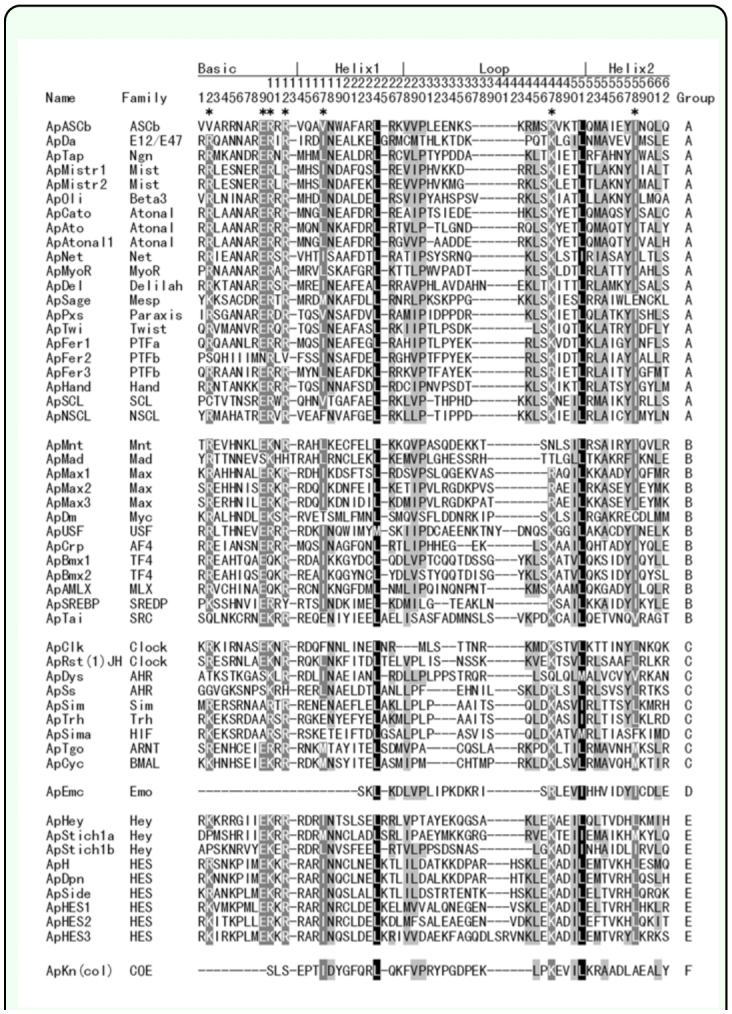
Alignment of 54 ApbHLH members. Designation of basic, helix 1, loop, and helix 2 follows Ferre-D'Amare et al. (1993). The family names and high-order groups have been organized according to Table 1 in Ledent et al. (2002). Highly conserved sites are indicated with asterisks on the top. High quality figures are available online.

**Figure 2.**
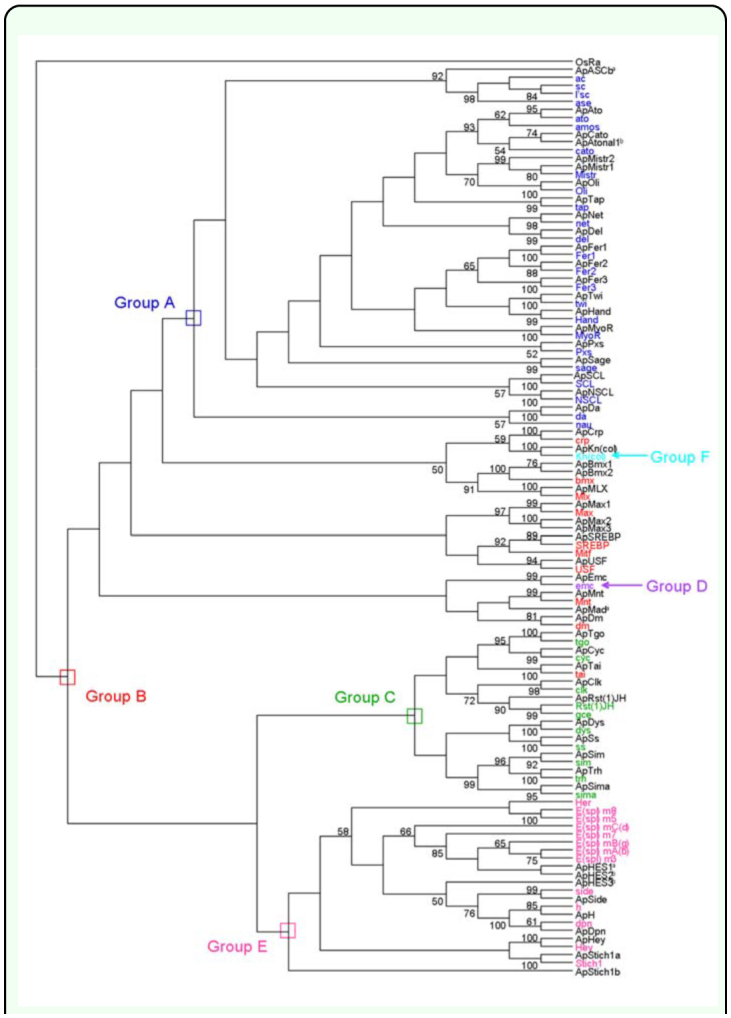
Phylogenetic relationship of 54 ApbHLH members with 59 *Drosophila melanogaster* bHLH members. A neighbor-joining (NJ) tree is shown. Bootstrap values less than 50 are not shown. The higher-order group labels are in accordance with Ledent et al. (2002). ApbHLH member marked with ^a^ or ^b^ meant that it did not form a monophyletic clade with any single D. *melanogaster* bHLH member and was subject to separate phylogenteic analyses with bHLH members from *Apis mellifera, Bombyx mori,* and *Tribolium castaneum.* High quality figures are available online.

The tblastn searches, sequence alignment, and examination of the 19 conserved amino acid sites revealed that there were 54 *bHLH* genes in *A. pisum* genome. The alignment of all 54 ApbHLH members is shown in Figure 1 and the phylogenetic tree constructed using amino acids from 54 ApbHLH motifs and 59 *D. melanogaster* bHLH motifs is shown in Figure 2. Figure 1 and 2 show that there were 21, 13, 9, 1, 9, and 1 ApbHLH members in group A, B, C, D, E, and F, respectively. In Figure 1, there are two most conserved sites located at sites 24 and 51 of the bHLH motif, respectively. Besides these, there are seven other sites that are also conserved (indicated with asterisks on top of Figure 1). Because the phylogenetic analyses have provided sufficient bootstrap support, the identified ApbHLH motifs were named according to nomenclature used by *D. melanogaster* bHLH sequences. In the case where one *D. melanogaster* bHLH sequence has two or more *A. pisum* homologues, the researchers used ‘a’, ‘b’, and ‘c’ or T, ‘2’, and ‘3’ etc to number them. For instance, two homologues of the *D. melanogaster Mist, Bmx* and *Stich 1,* genes were found in *A. pisum.* Therefore, these *ApbHLH* genes were named *ApMist1* and *ApMist2, ApBmx1* and *ApBmx2,* and *ApStich1a* and *ApStich1b,* respectively. Fiftyfour ApbHLHs were named in accordance with the corresponding *D. melanogaster* and other insect homologues as listed in Table 1.

### Identification of orthologous families

**Figure 3.**
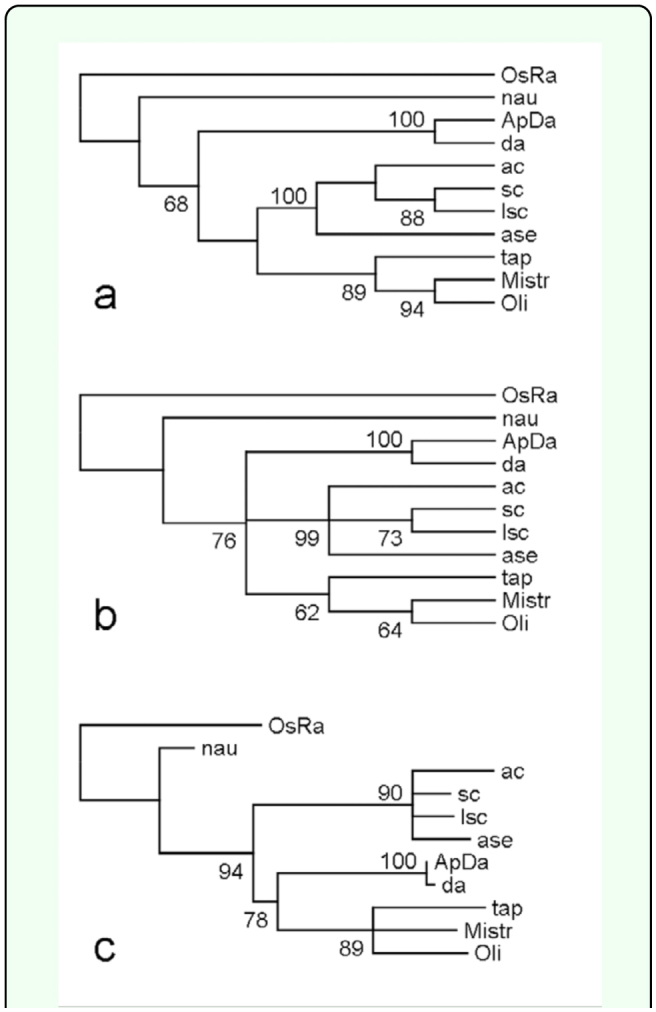
In-group phylogenetic analyses of ApDa. (a), (b), and (c) are NJ, MP, and ML trees, respectively, constructed with one *Acyrthosiphon pisum* bHLH member (ApDa) and seven group A bHLH members from *Drosophila melanogaster.* In all trees, OsRa (the rice bHLH motif sequence of R family) was used as the outgroup High quality figures are available

Ortholog identification has been very uncertain since there is no absolute criterion that can be used to decide whether two genes are orthologous (Ledent and Vervoort 2001). However, in previous studies (Wang et al. 2007, 2008) in-group phylogenetic analysis was adopted to identify homologues for the unknown sequences that would form a monophyletic clade among themselves. So a more certain criterion was used based on the criterion used by Ledent et al. (Ledent and Vervoort 2001; Ledent et al. 2002): If an unknown single *A. pisum* bHLH forms a monophyletic clade with another bHLH of known family in phylogenetic trees constructed with different methods, and all the bootstrap values exceed 50 then the known member will be regarded as a homologue of the unknown sequence. Figure 3, as an example here, shows NJ, MP, and ML phylogenetic trees constructed with one *A. pisum* bHLH member (ApDa) and seven group A bHLH members from *D. melanogaster.* In all three trees, ApDa formed monophyletic clade with Da (daughterless) specimens of *D. melanogaster* with all bootstrap values as 100. Therefore, ApDa was considered an ortholog of Da *D. melanogaster.* Similar in-group phylogenetic analyses were conducted for each of the identified *A. pisum* bHLH members. All the bootstrap values of constructed NJ, MP, and ML trees for each of the identified *A. pisum* bHLH members were listed in Table 1 without showing the correspondent constructed trees. Table 1 showed that the orthology of *A. pisum* bHLH members with *D. melanogaster* and other insect species can be divided into the following categories:

First, among all the 54 *A. pisum* bHLH members: 32 ApbHLH members have all the bootstrap values over 50 (54 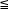 bootstrap values 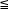 100) in constructed NJ, MP, and ML trees except *ApMax3* of which the bootstrap value of the MP tree is 42. These 32 ApbHLHs are *ApDa, ApMistr1, ApMistr2, ApOli, ApNet, ApMyoR, ApDel, ApTwi, ApFer1, ApFer3, ApHand, ApSCL, ApNSCL, ApMnt, ApMax1, ApMax2, ApMax3, ApCrp, ApMLX, ApSREBP, ApTai, ApClk, ApDys, ApSs, ApSim, ApTrh, ApSima, ApTgo, ApEmc, ApStich1a, ApSide,* and *ApKn(col).* The researchers have sufficient confidence to define the orthology of these ApbHLH motifs as corresponding to *D. melanogaster* bHLH orthologs.

Second, 5 ApbHLH members (namely *ApTap, ApFer2, ApDm, ApUSF,* and *ApBmx2*) have bootstrap values ranging from 77 to 99 in NJ and MP trees, except *ApDm* of which the bootstrap value of the MP tree is 45. In NJ and MP trees, each of them formed a monophyletic clade with the same *D. melanogaster* bHLH orthologue. However, they formed monophyletic clades (bootstrap value:58
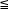
bootstrap values
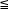
89) with other *D. melanogaster* bHLH members in ML trees. Specifically, the orthologue *of ApTap* was *tap* of *D. melanogaster* in NJ and MP trees, but was *cato* in ML trees. The orthologue of *ApFer2* was *Fer2* of *D. melanogaster* in NJ and MP trees, but was *Pxs* in ML trees. The orthologues of *ApDm, ApUSF,* and *ApBmx2* were *dm, USF,* and *bmx* of *D. melanogaster,* respectively, in NJ and MP trees, but all were *SREBP* in ML trees. The orthology for these 5 ApbHLH members has been defined according to the statistical support from NJ and MP trees.

Third, 7 ApbHLH members (namely *ApAto, ApSage, ApPxs, ApBmx1, ApHey, ApStich1b,* and *ApH*) formed monophyletic clades with bootstrap values ranging from 52 to 100 in NJ and MP trees, but did not form any monophyletic groups with any single bHLH sequence in ML trees (marked with n/m* or n/m in Table 1). Four other ApbHLH members (namely *ApCato, ApRst(1)JH, ApCyc,* and *ApDpn*) formed monophyletic clades with bootstrap values ranging from 45 to 96 in one of the NJ, MP, and ML trees, but did not form any monophyletic clades in the other two trees. Although these 11 ApbHLH members did not have sufficient bootstrap support, the orthologs were defined because they each have one or two bootstrap supports to testify to their orthology to the correspondent *D. melanogaster* ortholog. This phylogenetic divergence of bHLH motif sequences between *A. pisum* and *D. melanogaster* probably means that these two insect species have evolved in quite different circumstances.

**Figure 4.**
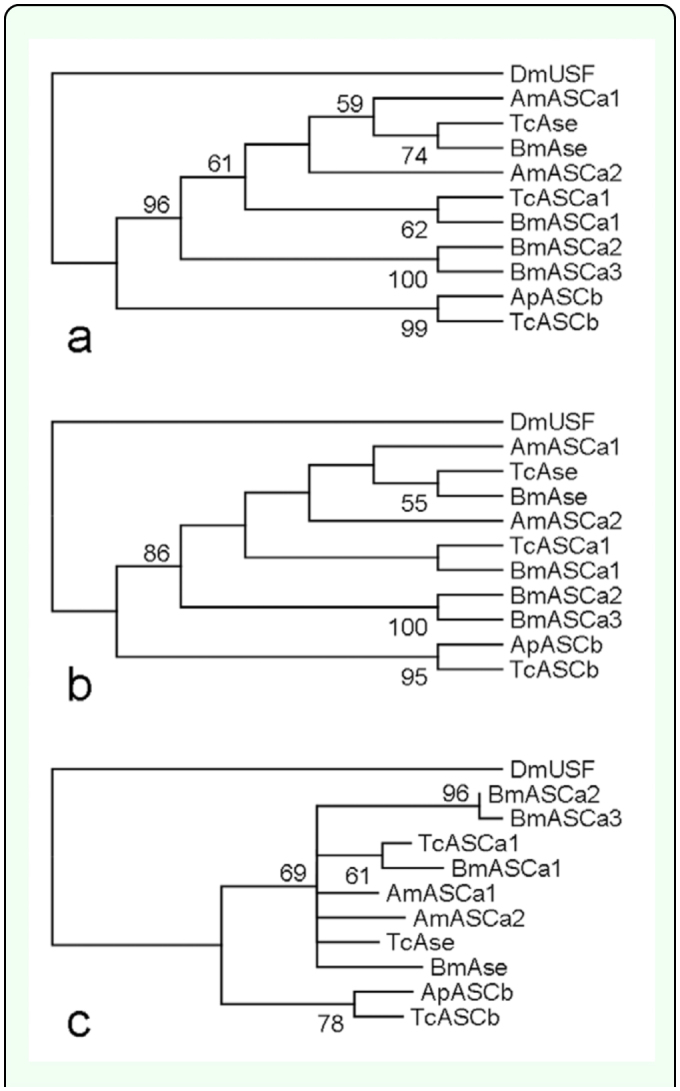
In-group phylogenetic analyses of ApASCb. (a), (b), and (c) are NJ, MP, and ML trees, respectively, constructed with one *Acyrthosiphon pisum* bHLH member (ApASCb) and nine ASC family members from *Apis mellifera, Bombyx mori,* and *Tribolium castaneum.* In all trees, bHLH motif sequence of DmUSF (*Drosophila melanogaster* upstream stimulation factor) was used as the outgroup. High quality figures are available online.

Finally, there are 6 ApbHLH sequences which did not form monophyletic clade with any *D. melanogaster* bHLH sequence in all constructed phylogenetic trees. They are *ApASCb, ApAtonall, ApMad, ApHES1, ApHES2,* and *ApHES3* (marked with ^a^ or ^b^ in Table 1 and Figure 2). Each of them was used to conduct in-group phylogenetic analyses with corresponding sequences from 3 other insect species, namely *A. mellifera, B. mori,* and *Tribolium castaneum.* For example, Figure 4 shows that ApASCb formed a monophyletic clade with TcASCb with bootstrap values ranging from 78 to 99. Therefore, it was considered an ortholog of TcASCb. Similarly, ApMad was found to be an ortholog of TcMad with all bootstrap values at 100 (Table 1). Orthology *of ApHES1* could also be defined, although the bootstrap values were not sufficiently high (35 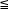 bootstrap values 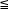 44) and no monophyletic calde was formed in two phylogenetic trees constructed. Orthology of *ApHES2, ApHES3,* and *ApAtonall* were the least clear. It was evident that *ApHES2* and *ApHES3* belonged to the H/E(spl) family. *ApAtonal1* was clearly a member of the Atonal family. Therefore, they have been named numerically (Table 1).

### Identification of protein sequences and genomic contigs

Protein sequence accession numbers for all the identified ApbHLH motifs are listed in Table 1. There are 3 ApbHLH motifs, of which, protein sequence accession numbers were not found in any protein databases. They are ApSREBP, ApDys, and ApFer2, respectively. Protein sequence accession numbers for 14 ApbHLH motifs were only found in the ‘*Ab initio* protein’ database in which all protein sequences were predicted from their corresponding genomic sequences. ApCyc protein sequence accession number was found in ‘RefSeq protein’ database. The rest of the ApbHLH protein sequences accession numbers were found in ‘Non-RefSeq protein’ database.

The coding regions and intron analysis for 54 *A. pisum* bHLH motifs are listed in Table 2. These data indicate that there are 26 ApbHLH members with introns in their bHLH motifs, and the total number of intron is 34. Eighteen ApbHLH members have one intron, among which *ApDa, ApClk, ApTgo, ApCyc, ApStich2,* and *ApHES1* have introns in the basic region; *ApMistr1, ApMistr2,* and *ApPxs* have introns in helix 1 region; *ApASCb, ApUSF, ApCrp, ApBmx1,* and *ApSREBP* have introns in the loop region; and *ApSage, ApSCL, ApMnt,* and *ApBmx2* have introns in helix 2 region. Eight ApbHLH members have two introns, among which *ApH, ApDpn, ApSide1, ApSide2, ApHES3,* and *ApKn(col)* have introns in the basic and loop regions, *ApTai* has introns in the basic and helix 2 regions, and *ApMad* has introns in the loop and helix 2 regions. The longest intron in the *A. pisum* bHLH motif is 30,718 bp (base pairs), and the average length of intron is 4193 bp. Compared with other insect species, the number and length of introns are remarkably higher in *A. pisum.* For instance, in the *B. mori* and *Apis mellifera* bHLH motifs, there are only 12 and 9 introns with the longest introns being 7083 bp and 4460 bp, and the average length of introns being 1352 bp and 1326 bp, respectively. Also, 8 ApbHLH motifs have two introns, while no bHLH motif has been found to have two introns in *Bombyx mori* and *A. mellifera* (Wang et al. 2007, 2008).

## Conclusion

Our study identified 54 bHLH members in the *A. pisum* genome. All ApbHLH members have been defined by their names and families according to various phylogenetic analyses with bHLH homologues of *D. melanogaster, A. mellifera, B. mori,* and *T. castaneum.* Among all ApbHLH members, 48 ApbHLH members have homologues in *D. melanogaster,* and 3 ApbHLH members have homologues in *B. mori* and *T. castaneum.* Three ApbHLH motifs' protein sequence accession numbers were not found in any protein database. The researchers also found that the number and average length of introns in ApbHLH motifs are higher than those in other insect species, which is quite possibly the consequence of the insertion of increased numbers of transposable elements in the coding regions of ApbHLH proteins as revealed by the International Aphid Genomics Consortium (2010). The above results would provide useful background information for future studies on functions of bHLH proteins in the regulation of *A. pisum* development.

**Table 1.**
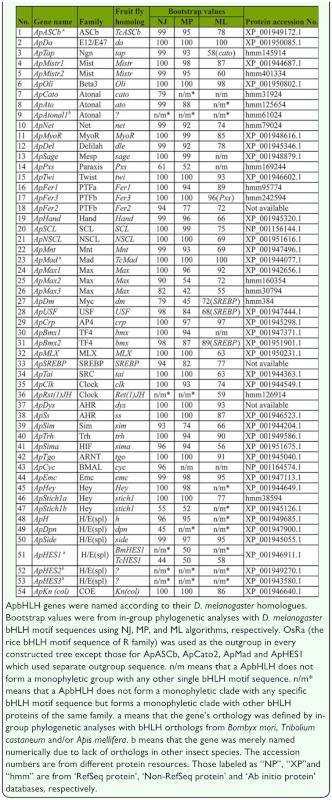
A complete list *of Acyrthosiphon pisum* bHLH genes.

**Table 2.**
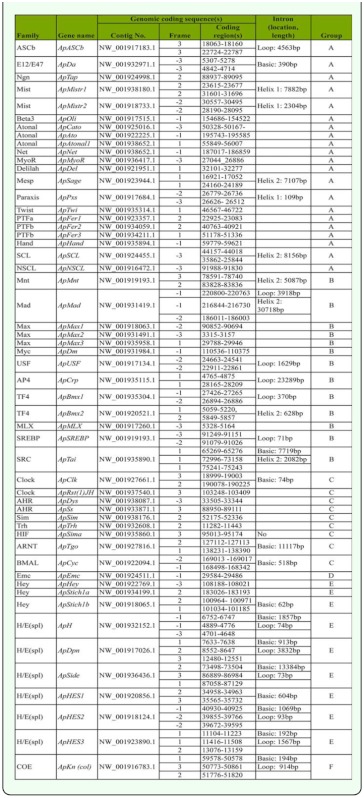
Table 2. Coding regions, intron location and length of 54 ApbHLH motifs.

## Abbreviations:

**Ap**,: *Acyrthosiphon pisum*;
**Am**,: *Apis mellifera*;
**Bm**,: *Bombyx mori*;
**Tc**,: *Tribolium castaneum*
